# Recent updates in mammalian oxylipin biochemistry

**DOI:** 10.1016/j.jbc.2025.110629

**Published:** 2025-08-26

**Authors:** Valerie B. O’Donnell

**Affiliations:** School of Medicine, Cardiff University, Cardiff, UK

**Keywords:** lipid, oxylipin, eicosanoid, cyclooxygenase, prostaglandin, lipoxygenase

## Abstract

Oxylipins represent large families of bioactive lipids generated through the oxygenation of fatty acids. Although originally discovered almost 100 years ago, significant interest in these lipids continues today with many important discoveries on their structures and functions still being made, particularly in regards to mammalian biology. This review will highlight some aspects of the current status of the field, focusing on recent findings that continue to challenge our views about how oxylipins are made and act in human health and disease. Areas to be covered include: an update on the ever-expanding roles of prostaglandinE_2_ in cancer, octadecanoid structures and nomenclature, complex forms of oxylipins, and challenges and considerations for oxylipin analysis including potential clinical utility.

The term oxylipin generally refers to oxygenated metabolites of primarily monounsaturated fatty acid and polyunsaturated fatty acid (PUFA) that originate mainly from linoleic acid (LA), arachidonic acid, eicosapentaneoic acid, and docosahexanoic acid and they can be either enzymatic or nonenzymatic in origin. The first use of “oxylipin” was in 1991 when Hamberg described the formation of vicinal dihydroxy fatty acids (FAs) in the red alga Gracilariopsis lemaneiformis (1). Since then it has become used as an umbrella term to encompass well-known families of bioactive oxygenated FAs, such as octadecanoids (C18) (2), eicosanoids (C20) (3, 4), docosanoids (C22) (5, 6), as well as prostaglandins (PGs) and leukotrienes. In the LIPID MAPS Classification and Nomenclature, these lipids are all grouped under the category fatty acyls, within main classes that refer to their hydrocarbon chain lengths (https://lipidmaps.org/databases/lmsd/browse). Oxylipins have been known about for almost 100 years, with early work during the 1930s by Kurzrock and Lieb (7), and von Euler (8), on PGs. Later, in the second half of the 20th century, PGs and thromboxanes were structurally characterized by Hamberg, Bergström, and Samuelsson (9, 10), leading to the awarding of a Nobel Prize. Since that time, there has been an explosion of research into the biology and biochemistry of this large and expanding group of lipids, which continues to show no sign of waning in interest. The purpose of this review is to focus on recent findings in the field of mammalian oxylipin research and highlight gaps in knowledge that remain to be addressed, as well as discuss their analysis for both research and clinical applications.

## The ever-expanding role of PGE_2_ in cancer

PGE_2_ was one of the first members of the PG family to be identified and characterized many years ago by Bergström, Sjövall, Samuelsson *et al.*(11, 12, 13). It is a product of microsomal prostaglandin E synthase (mPGES), acting on PGH_2_, itself a product of either cyclooxygenase-1 or cyclooxygenase-2 (COX). PGE_2_ is formed at varying levels depending on the specific biological context and signals potently at very low concentrations, binding and activating G protein–coupled receptors (GPCRs), termed EP1-4. This evokes a huge range of tissue and organ specific responses that are even still being delineated as our knowledge of how this important lipid participates in biology continue to be revealed.

PGE_2_ plays important and diverse roles in cancer, for example, through stimulating tumor growth and progression, promoting cell proliferation, preventing apoptosis, and increasing invasion and metastasis. These effects are mediated particularly through EP 1, 2 and 4 receptors, signaling through diverse mechanisms including PKC/NF-κB/Forkhead box protein C2, and EGFR/PI3K (14, 15, 16). A urinary metabolite of PGE_2_, PGE-M is elevated in human cancers including gastric and colon and correlates with poor outcomes (17, 18, 19), suggesting the lipid as a potential therapeutic target, as well as a biomarker (20). Since the early 2000s there has been huge interest in delineating how PGE_2_ promotes cancer and the involvement of PGE_2_-driven inflammation in this process. Major efforts were made to develop selective inhibitors of its signaling, as summarized in a recent review (21). Initially, based on compelling population level data, nonsteroidal anti-inflammatory drugs (NSAIDs) and aspirin were trialed, however due to their potential for side effects, including gastrointestinal bleeding, this has not made it into routine clinical use. A separate approach relates to inhibition of mPGES, to prevent generation of PGE_2_ directly, aiming to avoid the cardiovascular impacts of NSAIDs such as higher blood pressure due to prostacyclin inhibition. Although there is interest in development of mPGES inhibitors, only two phase I studies are listed on clinicaltrials.org, looking at evaluating safety, tolerability, and pharmacokinetics in healthy and elderly subjects of a drug, GRC27864 (NCT02361034, NCT02179645), and no studies in cancer are listed. These completed already in 2014/15, and showed good safety and efficacy *in vivo* (22), but follow on trials do not seem to have been initiated since.

As a more targeted approach, EP2 and EP4 antagonists are being actively tested both for their ability to slow gastric cancers and modulate immune responses. As yet, these have not made it into clinical use, although several trials are ongoing. An attractive hypothesis for this therapy relates to the combined use of EP receptor antagonists with immune checkpoint inhibitors. This takes into account how PGE_2_ acts in the immune system, for example, through contributing to immune evasion by recruiting regulatory T cells (Tregs) to the tumor microenvironment (23). In this context, Tregs can suppress the ability of cytotoxic T cells (CD8+ T cells) from targeting cancer cells. PGE_2_ also regulates macrophages, altering their polarization toward immunosuppression (24). As an example of a potential mechanism by which inflammation and PGE_2_ might influence colon cancer development, recent studies have shown how the lipid disrupts gut microbiota Treg communication *via* EP4 to increase inflammation, with this dysregulated in aging (25, 26).

**Figure 1:**
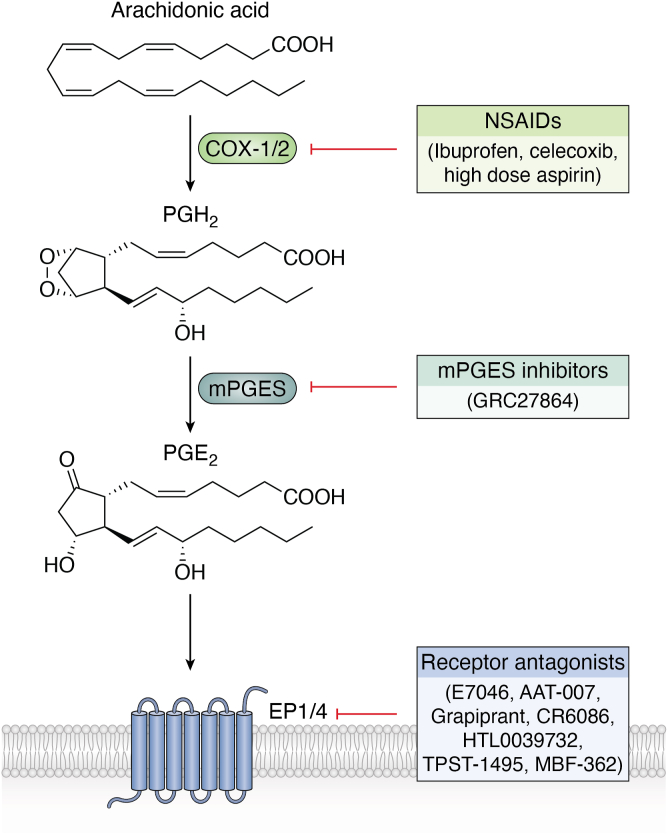
**Strategies for preventing signaling actions of PGE_2_ in cancer.** PGE_2_, prostaglandinE_2_.

Six trials on the EP4 antagonist E7046/AN0025 are listed on clinicaltrials.gov. Two are terminated/suspended and two are active (NCT05191667, NCT04432857, NCT04975958, NCT02540291). In 2020, this drug was found to be safe and well tolerated in patients with various advanced tumors (27), and a trial in advanced rectal cancer in combination with chemoradiation completed in 2021 reporting encouraging preliminary efficacy results, and aiming to recommend phase 2 doses (NCT03152370) (28, 29). However, a trial that commenced in 2024 evaluating efficacy of this drug by progression free survival and other outcome measures appears to have been withdrawn (NCT05358691). A phase II trial of another EP4 antagonist (AAT-007) in advanced solid tumors was also withdrawn, this time due to the principal investigator moving institutions (NCT02538432), while a phase Ib study on the EP4 antagonist Grapiprant in combination with immune checkpoint inhibition completed in 2023 but has not yet reported (NCT03658772). Other studies on EP4 antagonists remain open and recruiting including a phase Ib/IIa study on CR6086 (NCT05205330), and a phase I/IIa study on HTL0039732, both in combination with immune checkpoint blockade (NCT05944237). Encouragingly, a dual EP2/EP4 antagonist was superior at reducing tumor burden in multiple mouse models to targeting EP4 alone (30). This approach is now being trialed in humans with a trial of a dual EP2/EP4 antagonist (TPST-1495) (NCT04344795) currently active with preliminary data reporting “pharmacodynamic activity, a manageable safety profile, and a potential signal of activity consistent with the preclinical data,” and an endometrial cancer therapy combination study is currently recruiting (NCT06129604) (31). Encouragingly, a phase II trial on this drug in familial adenomatous polyposis is about to open (NCT06557733), while another dual EP2/EP4 antagonist (MBF-362) recently completed a phase I evaluation for safety and preliminary efficacy in solid tumors (NCT05940571). Last, a study testing a triple antagonist of EP2, EP4, and the PGD2 receptor DP1 as monotherapy and in combination with immune checkpoint inhibition is recruiting (NCT06789172). This large suite of trials indicates significant pharma interest in the potential for PGE_2_ blockade in cancer, and over the next few years as phase II studies start to report, it will become clear whether there is a role for inhibition of EP antagonism in chemotherapy, particularly in combination with immune checkpoint blockade. Importantly, targeting EP receptors should avoid the side effects of NSAIDs. So far, urinary PGE-M has not become a routine biomarker for cancer diagnostics, but if EP antagonists prove clinically utility, then this may become a tool for patient stratification for therapies targeting PGE_2_. A summary of the strategies for blocking PGE2 formation and action in cancer is shown (Fig. 1).

## Revisiting old lipids, in the form of octadecanoids

**Figure 2:**
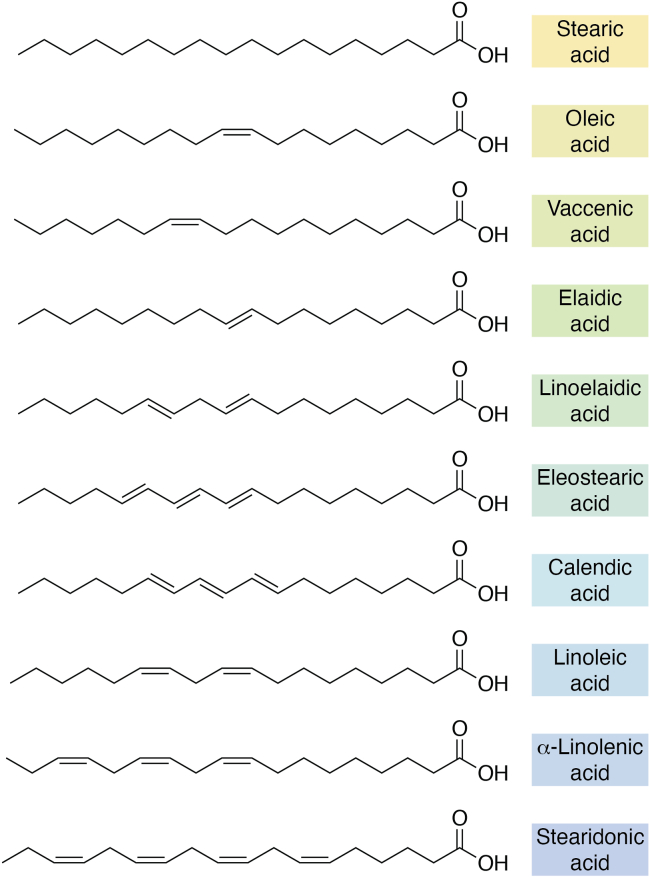
**Structures of various octadecanoids, showing positions of double bonds and geometric isomers**.

Octadecanoids are oxylipins derived from oxygenation of C18 PUFA, most commonly linoleate (LA, 18:2,ω6) and α-linolenate (18:3,ω3) (Fig. 2). Although much is known regarding their formation and function in plants (32), where they are essential for stress, growth, and development, less research has been undertaken into their roles in mammalian health and disease (33). Mono-hydroxy oxylipins derived from C18 PUFA are highly abundant in human tissues, with their plasma and serum levels being greater than those from other FA (34). Whether the lipids are formed during coagulation is not clear with one study showing that levels of oxylipins from LA increase significantly (35) in particular the 9- and 13-hydroxyoctadecanoids (HODE), from LA, while others showing little change (36, 37, 38). Currently, the sources of individual octadecanoids in blood are not clearly defined but could include circulating vascular cells, endothelium or organs such as liver, and enzymes that include both lipoxygenases (LOXs) (39) and COXs (40, 41). The best studied mammalian octadecanoids are CYP-derived epoxides and their corresponding diols which have been found to play roles in cardiac failure (42), adult respiratory distress syndrome (43), and regulation of vascular, cardiac, pulmonary, and renal health (reviewed in (44)). Since 2016, these lipids have been linked with skin physiology, pain, itch, FA uptake and transport, inflammation, and burn injury (45, 46, 47, 48, 49, 50, 51, 52). Increasing their levels *in vivo* reduces disease in various vascular disease models (53, 54, 55), suggesting they may play a role in dampening inflammation (52). In common with many other FA-derived oxylipins, monohydroxy, and epoxy octadecanoids are well known as low-affinity peroxisome proliferator–activated receptor γ ligands, and inducers, contributing to dampening inflammation including through suppression of NLR family pyrin domain–containing 3 inflammasome (56, 57, 58, 59, 60). This was first observed many years ago (60), and later shown to be how octadecanoids prevent lipopolysaccharide-driven inflammation (61). Thus, peroxisome proliferator–activated receptor γ activation/induction may largely explain how these lipids mediate vascular protective effects in many other models. Despite the large abundance of octadecanoids, in particular the HODEs, there remain many unanswered questions relating to their biological sources and signaling actions. Few specific GPCR for octadecanoids are yet known, with 9-HODE/GPR132 being the only International Union of Basic and Clinical Pharmacology recognized ligand binding pair, and with none recognized for epoxyeicosatrienoic acids (EETs), hydroxyoctadecanoic acids (HOMEs), or other octadecanoids (62). Low-affinity ligand binding has been claimed for several EETs and for 10-HOME with GPR40 (63, 64), although this is unlikely to be a truly specific GPCR-dependent signaling mechanism. In line with this, GPR40 is only recognized by International Union of Basic and Clinical Pharmacology as a free FA, but not octadecanoid receptor. 12,13-diHOME can increase calcium influx by TRPV1 in neurons, however as this is mediated *via* PKC activation at physiological lipid concentrations, it does not appear to due to direct receptor-ligand binding (65). Nitrated octadecanoids signal through their ability to participate in Michael additions with reactive thiol groups on proteins and this has been found to mediate diverse signaling actions that are overall anti-inflammatory in nature (66). Relating to skin physiology, an essential role for linoleate (including *via* retroconversion from arachidonic acid) in barrier formation has been known about since the 1980s (67, 68). Later, around 2010, several studies, including with KO mice, demonstrated a key role for LOXs in this process, as well as providing *in vivo* evidence of roles for 12R-LOX, eLOX3, and SDR9C7 (69, 70, 71, 72, 73). More recent studies provided the structures of the octadecanoid-containing ceramides in full (74, 75).

The last few years has seen a resurgence in interest in octadecanoid mammalian biology, in part driven by development of new analytical approaches (76) and recent updates to their nomenclature and classification, both of which serve to highlight the diversity of these compounds and how little is known about them (77). Chiral methods enabled up to 103 standards to be separately analyzed, revealing approximately 20 of these to be detectable and quantifiable in human and murine plasma (78). A protocol for this was recently published here (79). Last, desorption electrospray ionization mass spectrometry–multiple reaction monitoring imaging was recently applied to lung tissue LA and α-linolenate metabolites (80). These new analytical tools for the study of octadecanoids will undoubtedly help to stimulate further research into their biological roles. Highlighting the major gaps in this field, a comprehensive review into octadecanoid biology and bioactivity published in 2025 (77) concluded that many gaps exist in the field including developing a consensus on tissue distribution and endogenous concentrations, what specific receptors might exist, their triggers for biosynthesis, and delineating the structures and functions of lesser known octadecanoids. With the new analytical tools available, these questions are ripe for further exploration in the years to come.

## Oxylipins as part of larger molecules, expanding bioactions

Although oxylipins are traditionally considered to be generated and to mediate their bioactivity as short-lived, locally acting free acid autocoids, it has become increasingly recognized that they also form through biochemically regulated processes attached to larger lipids such as phospholipids, endocannabinoids, glycerides, acylceramides, and sterol esters, and that these species serve distinct functions to their free acid counterparts. Enzymatic formation is very different from the free radical–dependent formation of oxidized phospholipids that was the subject of intense research in the 1980s to 1990s, instead involving either direct oxidation (phospholipids, endocannabinoids, sterol esters) or esterification of an endogenously formed oxylipins by the action of Lands cycle enzymes (phospholipids). In the case of phospholipids, acute activation of 5- or 12-LOXs and COX-1 or COX-2 in immune cells and platelets leads to endogenous generation of oxylipins that are rapidly esterified into phospholipids, in substantial amounts, termed enzymatically oxidized phospholipids (eoxPLs) (81, 82). In contrast, 15-LOX1 can directly oxygenate phospholipids in macrophages and eosinophils (83, 84). A more recently identified pathway in platelets involves formation of a 12-hydroxyeicosatetraenoic acid-lysophosphatidylcholine (12-HETE-lysophosphatidylethanolamine) by direct action of 12-LOX (85). This follows generation of the 2-arachidonyl-lysophosphatidylethanolamine substrate by calcium-independent phospholipase A2 (85). A very different mechanism for eoxPL formation involves incorporation of exogenously added oxylipins into membranes in cells *in vitro*. This was extensively researched in the 1990s, for both EETs and HETEs, and it was suggested that the lipids might represent a potential reservoir for oxylipins to be later released through hydrolysis. However, the oxylipin incorporation pattern is different for endogenous oxylipins (summarized here (81)) and a biological role for oxPL formed through such transcellular mechanisms still has not been yet proven. In contrast, eoxPL generated endogenously by platelets were shown to support coagulation and thrombosis *in vitro,* and *in vivo* in mouse models, through enhancing the bioactivity of phosphatidylserine (86, 87, 88, 89). Current gaps in our knowledge about eoxPL include exactly which Lands cycle enzymes are responsible for their generation, including the acyl-CoA synthetase long-chain family members that generate the CoA intermediates, and how this process is regulated during inflammation and innate immunity. In this regard, a role for lysophosphatidylcholine acyltransferase-3 in selectively forming 12-LOX–derived diacyl-12-HETE-phosphatidylethanolamine in platelets was recently reported (82). In contrast, lysophosphatidylcholine acyltransferase-3 played no role in esterification of COX-1–derived 11-HETE or 15-HETE into platelet phospholipid pools, and the enzymes responsible remain so far unknown (82). Recently increased blood cell eoxPL were found to be related to elevated thrombotic risk, in rheumatoid arthritis (RA) and atherosclerotic cardiovascular disease (82, 90). In RA, this was driven by interleukin-6–dependent platelet activity in mice and associated with higher levels of anti-eoxPL-IgG in human RA (90).

In the case of endocannabinoids, direct oxygenation by COX-2 was reported many years ago, forming PG, thromboxane, and prostacyclin forms of glycerol esters and ethanolamides (91), some of which can interact with EP receptors (92). While there have been some reports on the bioactivity of these lipids over the years, including an anti-inflammatory role for glyceryl-PGD_2_ (93), a lot remains to be discovered relating to their mechanisms of formation and possible roles in other conditions where endocannabinoids are important. Esterified oxylipins were reported to be formed in plasma, including in lipoproteins where they accounted for >90% of the total oxylipins detected (94). Whether they are from autoxidation or enzymatic activity is not known but considering that this pool includes isoprostanes, it is possible that they are largely generated nonenzymatically. As a cautionary note, a more recent study showed their artefactual formation can occur during sample processing (95) (see here for a comprehensive review (96)). In plasma, the main source of esterified oxylipins appears to be glycerides, and also phospholipids. Relevance to disease was recently shown with significant changes seen in plasma from obese type 2 diabetics, where a potential involvement of 12-LOX was proposed based on positional isomer composition (95).

## Challenges in oxylipin analysis for basic research and clinical utility

The analysis of oxylipins is best performed using high sensitivity LC-MS/MS, with around 200 oxylipin primary standards now being available commercially, as well as several deuterated forms. State-of-the-art LC-MS/MS methods can robustly quantify down around 0.1 to 1 pg on column, depending on the platform, and many studies have been published on their application to oxylipin analysis in diverse tissues including plasma. Although the basic principles of oxylipin analysis is not any different from analysis of any other biomolecules, there are some additional points that researchers need to be aware of. These relate to isomeric separation, unusual behavior of particular oxylipins on column, and potential for artefactual generation during sample processing. These were recently summarized in a set of recommendations published by an International Lipidomics Society Interest Group, which was coauthored by almost 100 analysts working in the field (97). The recommendations described are for basic research, not clinical purposes, where a far higher degree of validation is required. Indeed, oxylipin analysis has not yet made it into routine clinical use. So far a clear demonstration that a specific oxylipin would represent a useful biomarker is lacking, considering that (i) increases in some, for example, PGs, may be a general feature of most forms of inflammation, and so may not provide further specificity than existing markers such as C-reactive protein, (ii) there are challenges with standardizing their analysis across clinical laboratories with significant potential for artefactual generation during sample processing (*e.g.* platelet-derived 12-HETE or thromboxaneB2 which can form during venipuncture). A recent study which avoided sampling artefacts reported plasma 12-HETE correlated with disease severity in COVID-19 (98). This may indicate a signature of platelet activation, but whether 12-HETE could indicate clinical need for anticoagulation therapy has not yet been tested. Consistent with this, plasma P-selectin is elevated during thromboembolism in COVID-19 (99). If true, this suggests that analysis of platelet activation markers, including oxylipins, may have future clinical utility in guiding the need for thrombolytic intervention.

Urinary oxylipins were the focus of much research during the 1980s and 1990s since they do not suffer from sampling artefacts, and the PGE_2_ metabolite, PGE-M was reported to be increased in various cancers, as outlined earlier in this review. Recently, Milne *et al.* turned their attention to the analysis of glucuronide metabolites that might represent endogenous markers of oxylipin biosynthesis, particularly where the primary oxylipin is low or has been rapidly metabolized. Glucuronide metabolites of isoprostanes were easily detected and characterized in human urine and could be altered through dietary modification (100). In contrast, although liver microsomes were able to metabolize resolvinD1 and D5 to glucuronides *in vitro* (101), these forms have not yet been detected in urine or other tissues. In summary, during the last several years, rapid advances in LC-MS/MS instrumentation and widening availability of primary and deuterated standards has enabled oxylipins to be studied in large numbers at low concentrations, in ways that were simply not possible up to now. An ongoing challenge in the field is to ensure consistency in their analysis, aiming for reproduction of key findings, to enable a consensus on their generation, mechanisms of formation and bioactions in health and disease.

## Conclusion

This review is intended as a short update to the field of mammalian oxylipin research. Overall, there is significant current interest in the clinical analysis of oxylipins, although in what context this will prove useful for diagnostics or therapeutics still somewhat needs to be defined. Anticoagulation or cancer therapeutics represent promising avenues considering the major problem of thrombosis in almost all forms of disease and the significant current investment in therapies targeting PGE_2_ in cancer. Novel metabolites of oxylipins, measured in urine are now being revisited with new forms being characterized. This is a welcome development considering sampling artefacts in plasma which do not apply to urine, and so, revisiting previous work that defined the urinary metabolites of several oxylipins would be a worthwhile endeavor for the field.

## Dedication

This review is written in honor of William (Bill) Smith, a true gentleman scientist who I knew to be one of the founding fathers of COX enzymology and biochemistry in the late 20th century. During the 1990s and early 2000s, I followed very closely his seminal papers on the mechanisms of COX peroxidase activity, which informed greatly my own studies into how nitric oxide is consumed by COX peroxidase, through acting as a direct reducing substrate. Following this his demonstration of COX homodimers was revolutionary in the field and went on to explain the specific interactions of various inhibitors and FAs with the different isomers. For about 4 years in the early 2010s, I would spend a month a year in Robert (Bob) Murphy’s lab in Denver, learning how to analyze oxylipins using LC-MS/MS. Due to their great friendship, Bill was often visiting at the same time, and so we overlapped somewhat, which I saw as a wonderful opportunity not to be missed, to expand my knowledge of COX enzymology. Those days are greatly missed.

## Conflict of interest

The authors declare that they have no conflicts of interest with the contents of this article.
